# Ring opening of 2-aza-3-borabicyclo[2.2.0]hex-5-ene, the Dewar form of 1,2-dihydro-1,2-azaborine: stepwise versus concerted mechanisms

**DOI:** 10.3762/bjoc.9.86

**Published:** 2013-04-18

**Authors:** Holger F Bettinger, Otto Hauler

**Affiliations:** 1 Institut für Organische Chemie, Universität Tübingen, Auf der Morgenstelle 18, 72072 Tübingen, Germany

**Keywords:** ab initio, azaborine, BN aromatics, Dewar isomer, reaction mechanism

## Abstract

The ring opening of the Dewar form of 1,2-dihydro-1,2-azaborine, 2-aza-3-borabicyclo[2.2.0]hex-5-ene (**3**) is investigated by theoretical methods by using multiconfiguration SCF (CASSCF) and coupled cluster theory [CCSD(T)] with basis sets up to polarised quadruple-zeta quality. The title compound was previously reported to form photochemically in cryogenic noble gas matrices from 1,2-dihydro-1,2-azaborine (**4**). Four reaction paths for the thermal ring opening of **3** to **4** could be identified. These are the conventional disrotatory and conrotatory electrocyclic ring-opening pathways where the BN unit is only a bystander. Two more favourable paths are stepwise and involve 1,3-boron–carbon interactions. The lowest energy barrier for the isomerisation reaction, 22 kcal mol^−1^, should be high enough for an experimental observation in solution. However, in solution the dimerisation of **3** is computed to have a very low barrier (3 kcal mol^−1^), and thus **3** is expected to be a short-lived reactive intermediate.

## Introduction

**Scheme 1:**
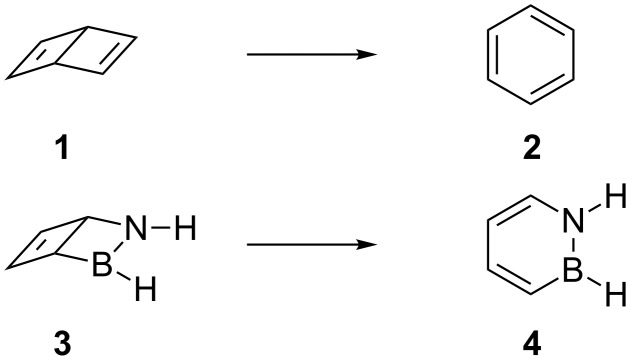
Isomerisation of bicyclo[2.2.0]hexa-1,3-diene, Dewar benzene (**1**), to benzene (**2**) and of 2-aza-3-borabicyclo[2.2.0]hex-5-ene (**3**) to 1,2-dihydro-1,2-azaborine (**4**).

The barrier for ring opening of Dewar benzene (**1**) to yield benzene (**2**) is high enough to give this benzene valence isomer a half life of about two days [1] at room temperature despite the significant exothermicity (60–70 kcal mol^−1^) of the isomerisation reaction (Scheme 1) [2–6]. The relatively high barrier (Δ*H*^‡^ = 25.1 ± 2 kcal mol^–1^) [7–8] is due to the fact that the formation of benzene from **1** would require a disrotatory ring opening that is orbital-symmetry-forbidden according to the Woodward–Hoffmann rules [9–11]. The allowed conrotatory electrocyclic opening of one of the cyclobutene moieties of **1**, on the other hand, would result in a highly strained *cis*,*cis*,*trans*-cyclohexa-1,3,5-triene (*trans*-benzene) isomer [10–11].

Computational investigations of the isomerisation have been performed to reveal mechanistic details [12–14]. The most sophisticated investigation [14] (multireference configuration interaction, energies based on complete active space self-consistent field geometries, MRCI//CASSCF) confirmed an earlier conclusion [13] that the conrotatory motion is the lowest energy pathway. It was also demonstrated that the conrotatory transition structure connects **1** and benzene directly, i.e., without the involvement of *trans*-benzene [14]. The barrier computed (32.6 kcal mol^–1^) is lower than that for the disrotatory pathway by 6.6 kcal mol^–1^ [14].

We have recently reported that the irradiation (λ = 254 nm) of 1,2-dihydro-1,2-azaborine (**4**), a boron-nitrogen heterocycle that is isoelectronic and isosteric with benzene [15], results in its Dewar isomer 2-aza-3-borabicyclo[2.2.0]hex-5-ene (**3**) under the conditions of cryogenic noble gas matrix isolation [16]. Under these experimental conditions (*T* < 35 K), the isomerisation back to **4** is not observed [16]. Can **3** exist outside of cryogenic matrices? To answer this question, we report here a computational investigation of important intramolecular and intermolecular decomposition pathways of **3**.

## Results and Discussion

### Ring opening of **3**

#### Conrotatory and disrotatory ring opening

**Figure 1:**
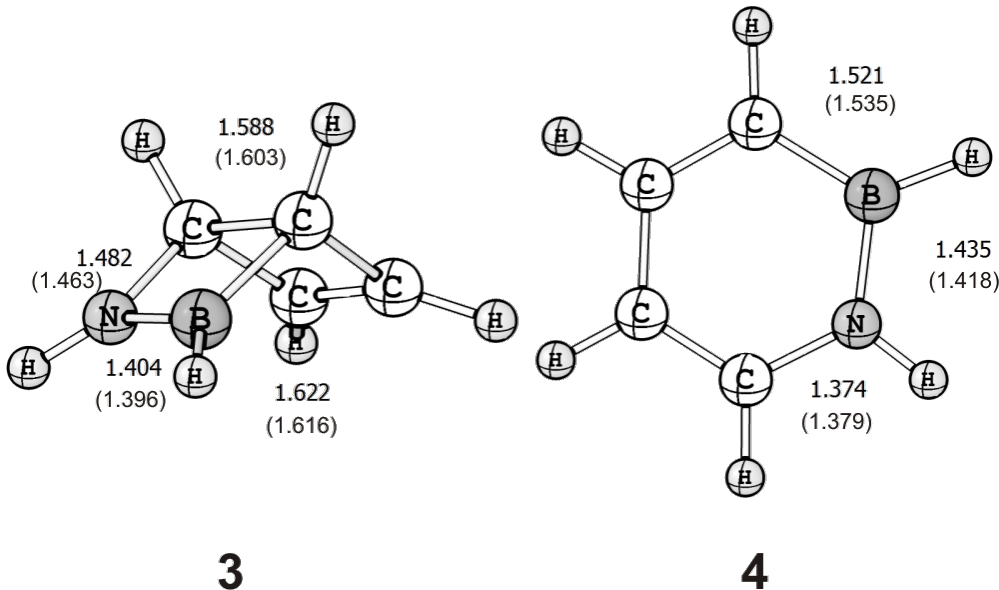
Geometries of **3** and **4** computed at the CCSD(T)/TZ2P and CASSCF(6,6)/6-31G* (in parentheses) levels of theory. Bond lengths are given in angstroms (Å).

**Figure 2:**
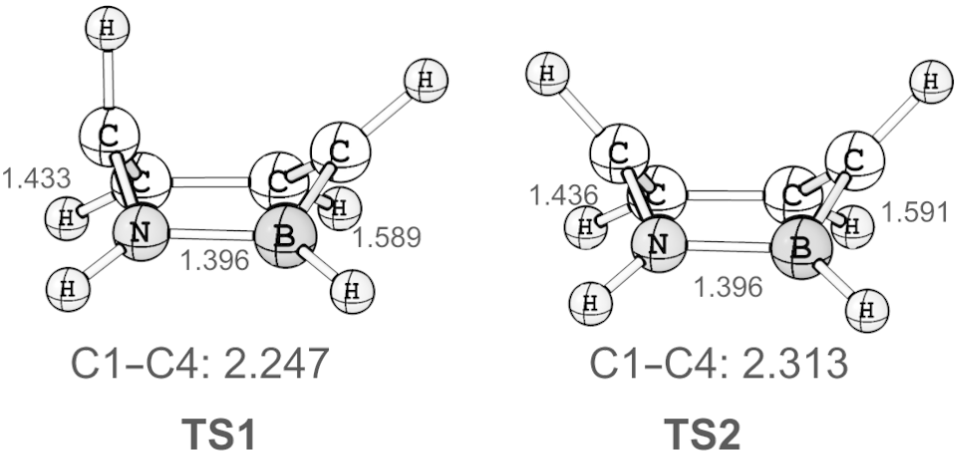
Geometries of **TS1** and **TS2** computed at the CASSCF(6,6)/6-31G* level of theory. C1–N, N–B, C4–B, and C1–C4 distances are given in angstroms (Å).

We have performed explorative computations using the CASSCF(6,6)/6-31G* method and could locate transition states for the conrotatory (**TS1**; *n*_imag_ = 1, *i*556 cm^−1^) and disrotatory (**TS2**; *n*_imag_ = 1, *i*628 cm^−1^) ring opening of **3**. Computation of the intrinsic reaction coordinates confirms that both **TS1** and **TS2** connect the Dewar form **3** to 1,2-dihydro-1,2-azaborine. These transition states are similar in geometry to those described earlier for the all-carbon system (see Figure 1 and Figure 2) [14]. The C1–C4 distance is shorter in **TS1** (2.247 Å) than it is in **TS2** (2.313 Å). The BN unit is a bystander in these two mechanisms as it is not involved in the ring-opening process.

The energies of these transition states were refined with multireference perturbation theory (MRMP2). In agreement with the results obtained for the all-carbon system [14], the barrier for the orbital-symmetry-allowed conrotatory ring opening is lower (26.5 kcal mol^−1^) than it is for the forbidden disrotatory reaction (30.1 kcal mol^−1^) (Table 1). The energy difference of about 4 kcal mol^–1^ is slightly smaller than that reported for the all-carbon system (7 kcal mol^−1^) [14]. Due to the use of different levels of theory (MRMP2 in the present work, MRCI by Havenith et al. [14]), the energy barriers for conrotatory ring opening of **1** and **3** cannot be directly compared. We have made no attempt to increase the level of theory for **TS1** and **TS2** beyond MRMP2, because we found two additional reaction paths that are significantly more favourable.

**Table 1:** Relative energies (*E*_rel_, in kcal mol^−1^ including zero-point vibrational energies, ZPVE) of 1,2-dihydro-1,2-azaborine (**4**), its Dewar valence isomer **3**, high energy minima, and the transition states for ring opening of **3** as computed at the MRMP2 and CCSD(T) levels of theory.

Compounds	*E*_rel_(MRMP2)^a^	*E*_rel_(CCSD(T))^b^

**3**	0	0
**4**		−59.3
**TS1** (conrotatory)	26.5	31.0^c^
**TS2** (disrotatory)	30.1	–
**MIN1**	–	20.3
**TS3**	–	21.7
**TS4**	–	25.8
**MIN2**	---	17.8
**TS5**	–	19.1
**TS6**	–	22.2

^a^MRMP2-CASSCF(6,6)/6-31G*//CASSCF(6,6)/6-31G*, ZPVE were obtained at CASSCF(6,6)/6-31G*; ^b^CCSD(T)/cc-pVQZ//CCSD(T)/TZ2P, ZPVE were obtained at CCSD(T)/DZP; ^c^ CCSD(T)/cc-pVQZ based on CASSCF(6,6)/6-31G*+ZPVE data for **TS1** and **3**.

#### Stepwise ring opening

**Figure 3:**
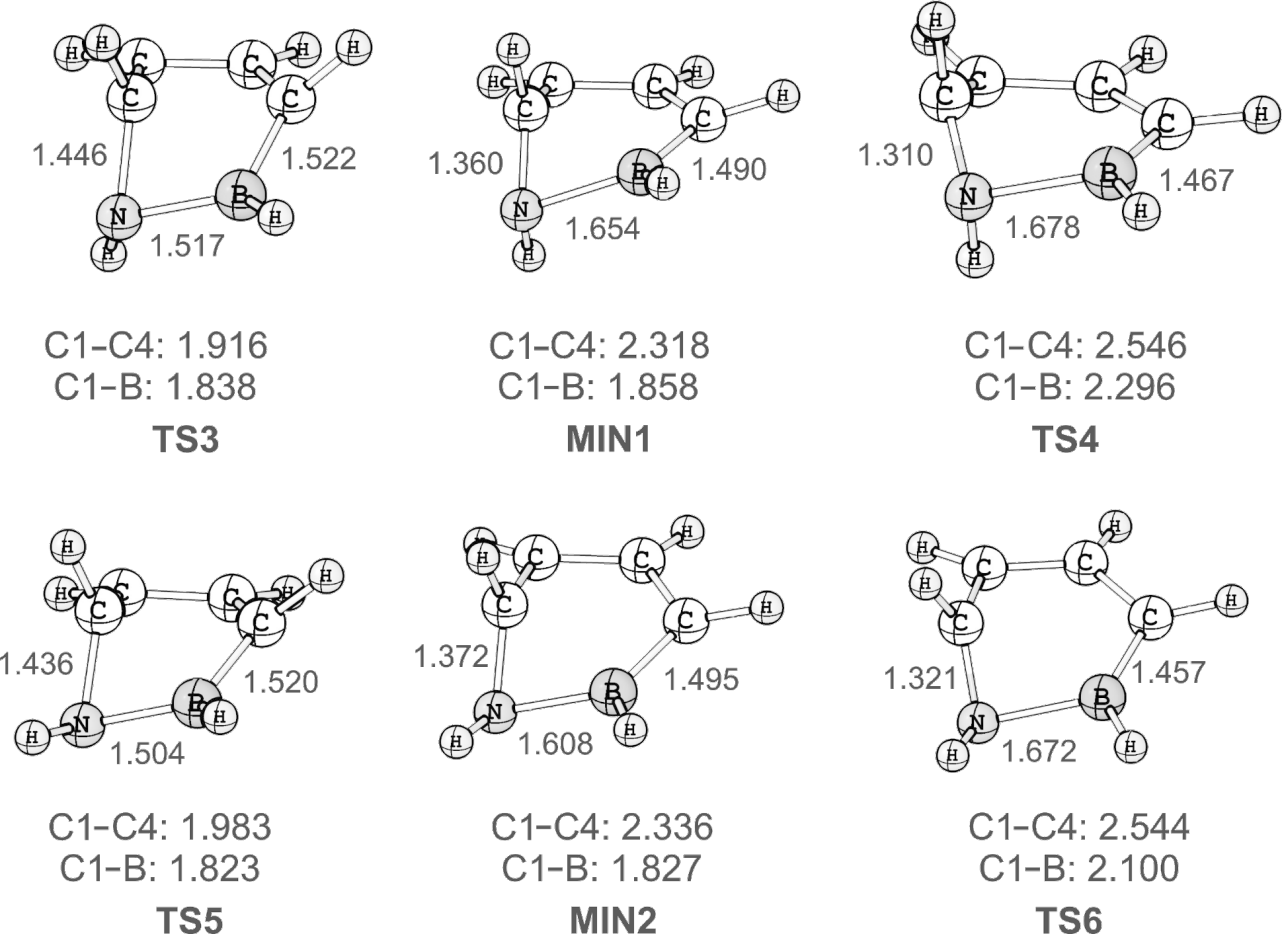
Geometries of **MIN1**, **TS3**, **TS4** and **MIN2**, **TS5**, **TS6** computed at the CCSD(T)/TZ2P level of theory. C1–N, N–B, C4–B, C1–C4, and C1–B distances are given in angstroms (Å).

Two additional pathways, both of them stepwise, for the ring opening of **3** could be identified. As the energies for the two reactions paths are very similar, we used coupled-cluster theory to obtain highly accurate structures (see Figure 3) and energies of the stationary points involved in ring opening. The CCSD(T) geometry optimizations arrive at a minimum on the potential-energy surface (**MIN1**). Characteristic of this intermediate **MIN1** is a short B–C1 distance of 1.858 Å, while the C1–C4 distance is increased to 2.318 Å. The nitrogen atom is strongly pyramidalised resulting in an H–N–C1–H dihedral angle of 175.2°. A second minimum **MIN2** between **3** and 1,2-dihydro-1,2-azaborine could also be located with the CCSD(T) method. The structure of **MIN2** is characterised by an almost ecliptic orientation of the N–H and C1–H bonds as the dihedral angle is only 7.8°. The B–C1 distance of 1.827 Å is slightly shorter, while the C1–C4 distance of 2.336 Å is slightly longer than in **MIN1**. Hence **MIN1** and **MIN2** mainly differ by the relative orientation of the N–H bond. The strong pyramidalisation of the nitrogen atom and the short B–C1 distance show that in these two reaction pathways the BN unit is no longer just a bystander. The mode of rotation that results in **MIN1** and **MIN2** may be considered conrotatory, but the C4H group has moved significantly more than the C1H group. The electron pair of the breaking C1–C4 bond is utilized for interaction with the boron centre. As a consequence, the nitrogen lone pair is more localised resulting in a pyramidalisation of the nitrogen centre.

At our highest level of theory, CCSD(T)/cc-pVQZ, **MIN2** is more stable than **MIN1** by 2.5 kcal mol^–1^. **MIN2** is 17.8 kcal mol^−1^ higher in energy than the Dewar form. Both **MIN1** and **MIN2** correspond to shallow minima on the potential energy surface. The barrier for collapse of **MIN1** to the Dewar form **3** through transition state **TS3** is only 1.4 kcal mol^−1^, while formation of 1,2-dihydro-1,2-azaborine from **MIN1** through **TS4** has a barrier of 5.5 kcal mol^−1^. Likewise, collapse of **MIN2** to the Dewar form **3** via **TS5** has a barrier of only 1.3 kcal mol^−1^, and formation of 1,2-dihydro-1,2-azaborine through **TS6** has a barrier of 4.4 kcal mol^−1^.

For most of the stationary points the T_1_ diagnostic [17], a measure of the reliability of single-reference based CCSD(T) theory, is below the critical value of 0.02 indicating that the CCSD(T) treatment should produce highly reliable results. Only for **TS4** and **TS6** are the T_1_ diagnostics 0.024 and 0.023, respectively. To confirm that the single reference CCSD(T) treatment produces satisfactory results also for these stationary points, we have computed the completely renormalised CR-CCSD(T)L energies for all species, as CR-CCSD(T)L has been shown to dramatically improve CCSD(T) results of multireference cases. The CR-CCSD(T)L energies are within 0.2 kcal mol^−1^ of the CCSD(T) values for all these species, including those (**TS4** and **TS6**) with slightly elevated T_1_ diagnostics.

Comparison of the energies of the stationary points along the stepwise pathways with **TS1** and **TS2** is hampered by problems associated with locating the latter at the CCSD(T) level. We have thus computed the barrier for conrotatory ring opening at the CCSD(T) and CR-CCSD(T) levels using the CASSCF(6,6) geometries. This shows that the stepwise mechanism is more favourable than the conrotatory opening by 9 kcal mol^–1^.

In summary, the energetically most favourable pathway for the ring opening of **3** to 1,2-dihydro-1,2-azaborine involves **MIN2** as a shallow intermediate and has a highest energy barrier of 22 kcal mol^−1^. This is roughly 10 kcal mol^−1^ lower than the lowest energy pathway for ring opening of Dewar benzene. Therefore, the lifetime of the Dewar form **3** is expected to be significantly lower than that of Dewar benzene. Nonetheless, with a barrier for isomerisation of about 22 kcal mol^−1^, **3** should be observable in solution.

### Dimerisation of **3**

Alternative pathways for disappearance of **3** may be provided by intermolecular reactions that are in principle feasible in solution. Such pathways are of particular importance, as the BN unit in **3** is an aminoborane (RHB=NHR) derivative. Aminoboranes with small substituents are unstable with respect to dimerisation or oligomerisation. In 1,2-dihydro-1,2-azaborine, such a dimerisation is not observed, probably due to the aromatic character of the six-membered ring. In **3**, however, this aromatic stabilisation is no longer available.

**Figure 4:**
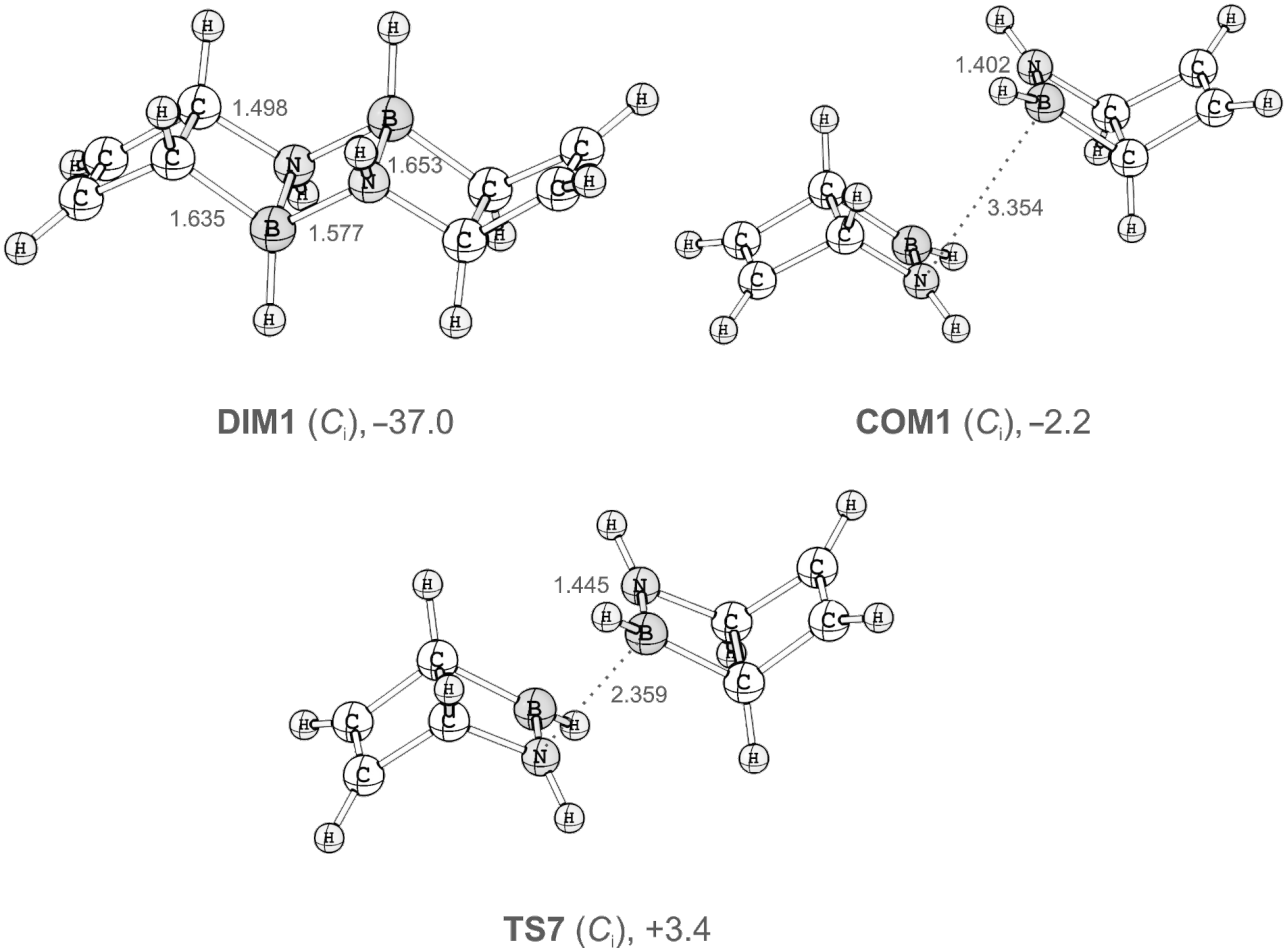
Geometries of **DIM1**, **COM1**, and **TS7** computed at the SCS-RIMP2/def2-TZVP level of theory. Distances are given in angstroms (Å). Energies (in kcal mol^−1^) relative to two separated molecules of **3** were obtained at the same level of theory and include ZPVE corrections obtained with the smaller def-SV(P) basis set.

For the sake of simplicity, we only considered dimers of **3** (see Figure 4 and Supporting Information File 1 for structures). All three diastereomeric dimers of **3** are thermodynamically more stable than two noninteracting monomers. Formation of the most stable dimer, **DIM1**, is favourable by 37 kcal mol^−1^. The barrier for its formation at the SCS-RIMP2/def2-TZVP level of theory is only 3.4 kcal mol^−1^ with respect to infinitely separated monomers, and 5.6 kcal mol^−1^ with respect to the energy of a van-der-Waals complex of two monomers.

## Conclusion

The following conclusions can be drawn from the computational investigation.

1. The “classical” conrotatory and disrotatory ring-opening reactions provide pathways for the isomerisation of **3** to 1,2-dihydro-1,2-azaborine. Similar to previous investigations of the ring opening of Dewar benzene (**1**), we find that the conrotatory pathway is lower in energy than the orbital-symmetry-forbidden disrotatory pathway. Both pathways are concerted.

2. In addition, two step-wise pathways that involve conformational isomeric minima were identified. Both of the minima have in common short 1,3-transannular C–B distances. The two pathways have very similar energy barriers (within 4 kcal mol^−1^). The more favourable one is lower in energy than the conrotatory ring opening by 9 kcal mol^−1^.

3. The lowest energy pathway for ring opening of **3** has a barrier of 22 kcal mol^−1^.

4. The lifetime of **3** in solution will not be limited by the ring opening to **4**, but rather by dimerisation. This is a strongly exothermic process that has a low barrier of 3 kcal mol^–1^ with respect to separated monomers. Thus, **3** is expected to be a highly reactive compound that will rapidly undergo dimerisation (or oligomerisation) reactions.

## Experimental

The active space in the CASSCF(6,6) computations included the π and π* orbitals for the 1,2-dihydro-1,2-azaborine system, while the four π and π* orbitals along with the C3–C6 σ/σ* orbitals were used for the Dewar form **3** and transition states **TS1** and **TS2**. Geometries were fully optimised and the nature of stationary points was confirmed by analytic computation of second derivatives. Intrinsic reaction coordinates were computed starting from the transition states by using the Schlegel–Gonzalez algorithm [18–19]. The same (6,6) active space was employed for the subsequent multireference second-order perturbation theory (MRMP2) [20] single-point-energy computations. All multireference computations employed the 6-31G* [21] basis set and were performed with the Gamess-US software [22].

The coupled-cluster method with single, double, and a perturbative estimate of triple excitations [CCSD(T)] [23] was employed for geometry optimisation by using analytic gradients [24] in conjunction with Dunning’s [25–26] DZP and TZ2P basis sets. Harmonic vibrational frequencies were computed by analytic second derivatives [27] using the DZP basis set to confirm the nature of stationary points and to obtain zero-point vibrational energies (ZPVE). The CCSD(T) gradient and Hessian computations were performed with CFOUR [28]. The CCSD(T)/TZ2P geometries were used for further energy refinement with Dunning’s [29] correlation consistent basis sets, cc-pVDZ, cc-pVTZ, and cc-pVQZ. These single point calculations were performed with the Turbomole program [30]. Its implementation of CCSD(T) uses integral-direct techniques and the resolution-of-the-identity approximation [31]. Therefore, the appropriate fitting basis set was chosen [32]. In addition, the so-called rigorously size-extensive completely renormalised coupled-cluster theory [CR-CC(2,3) or CR-CCSD(T)L] [33–34] was used in conjunction with the cc-pVDZ basis set using Gamess-US. The dimerisation of **3** was investigated by using Grimme’s spin-component-scaled MP2 method (SCS-MP2) [35], with the resolution-of-identity (RI) approximation for fast computations of two-electron integrals within the second-order Møller–Plesset perturbation theory (MP2) [36–37]. SCS-MP2 was shown recently to yield improved interaction energies compared to conventional MP2 [38–41]. The def-SV(P) [B/C/N: 3s2p1d; H: 2s] [42] and def2-TVZP [B/C/N: 5s3p2d1f; H: 4s2p1d] [37] basis sets in conjunction with the corresponding fitting bases were employed [37]. Harmonic vibrational frequencies were determined by using the def-SV(P) basis set by finite differences of analytic gradients and provided the zero-point vibrational energies (ZVPE).

## Supporting Information

File 1 Additional data.

## References

[1] van Tamelen E E, Pappas S P (1963). J Am Chem Soc.

[2] Volger H C, Hogeveen H (1967). Recl Trav Chim Pays-Bas.

[3] Oth J F M (1968). Recl Trav Chim Pays-Bas.

[4] Schäfer W (1966). Angew Chem, Int Ed Engl.

[5] Adam W, Chang J C (1969). Int J Chem Kinet.

[6] Oth J F M (1968). Angew Chem, Int Ed Engl.

[7] Breslow R, Napierski J, Schmidt A H (1972). J Am Chem Soc.

[8] Lechkten P, Breslow R, Schmidt A H, Turro N J (1973). J Am Chem Soc.

[9] Woodward R B, Hoffmann R (1969). Angew Chem.

[10] van Tamelen E E (1965). Angew Chem, Int Ed Engl.

[11] van Tamelen E E (1972). Acc Chem Res.

[12] Dewar M J S, Ford G P, Rzepa H S (1977). J Chem Soc, Chem Commun.

[13] Johnson R P, Daoust K J (1996). J Am Chem Soc.

[14] Havenith R W A, Jenneskens L W, van Lenthe J H (1999). J Mol Struct: THEOCHEM.

[15] Marwitz A J V, Matus M H, Zakharov L N, Dixon D A, Liu S-Y (2009). Angew Chem, Int Ed.

[16] Brough S A, Lamm A N, Liu S-Y, Bettinger H F (2012). Angew Chem, Int Ed.

[17] Lee T J, Taylor P R (1989). Int J Quantum Chem, Quantum Chem Symp.

[18] Gonzalez C, Schlegel H B (1989). J Chem Phys.

[19] Gonzalez C, Schlegel H B (1990). J Phys Chem.

[20] Hirao K (1993). Chem Phys Lett.

[21] Hariharan P C, Pople J A (1973). Theor Chim Acta.

[22] Schmidt M W, Baldridge K K, Boatz J A, Elbert S T, Gordon M S, Jensen J H, Koseki S, Matsunaga N, Nguyen K A, Su S (1993). J Comput Chem.

[23] Raghavachari K, Trucks G W, Pople J A, Head-Gordon M (1989). Chem Phys Lett.

[24] Scuseria G E (1991). J Chem Phys.

[25] Dunning T H (1970). J Chem Phys.

[26] Dunning T H (1971). J Chem Phys.

[27] Gauss J, Stanton J F (1997). Chem Phys Lett.

[R28] 28Stanton, J. F.; Gauss, J.; Harding, M. E.; Szalay, P. G. CFOUR, Coupled-Cluster techniques for Computational Chemistry, For the current version, see http://www.cfour.de

[29] Dunning T H (1989). J Chem Phys.

[R30] 30TURBOMOLE ,V6.4 2012, University of Karlsruhe and Forschungszentrum Karlsruhe GmbH, 1989-2007, TURBOMOLE GmbH, since 2007; available from http://www.turbomole.com

[31] Hättig C, Weigend F (2000). J Chem Phys.

[32] Weigend F, Köhn A, Hättig C (2002). J Chem Phys.

[33] Piecuch P, Kucharski S A, Kowalski K, Musial M (2002). Comput Phys Commun.

[34] Piecuch P, Wloch M (2005). J Chem Phys.

[35] Grimme S (2003). J Chem Phys.

[36] Weigend F, Häser M (1997). Theor Chem Acc.

[37] Weigend F, Häser M, Patzelt H, Ahlrichs R (1998). Chem Phys Lett.

[38] Hill J G, Platts J A, Werner H-J (2006). Phys Chem Chem Phys.

[39] Antony J, Grimme S (2007). J Phys Chem A.

[40] Takatani T, Sherrill C D (2007). Phys Chem Chem Phys.

[41] Bachorz R A, Bischoff F A, Höfener S, Klopper W, Ottiger P, Leist R, Frey J A, Leutwyler S (2008). Phys Chem Chem Phys.

[42] Schäfer A, Horn H, Ahlrichs R (1992). J Chem Phys.

