# Supplementary material for: Ring opening of 2-aza-3-borabicyclo[2.2.0]hex-5-ene, the Dewar form of 1,2-dihydro-1,2-azaborine: stepwise versus concerted mechanisms
Source: Beilstein J Org Chem. 2013 Apr 18;9:761–6. doi: 10.3762/bjoc.9.86 (PMC3678608; doi:10.3762/bjoc.9.86)
Supplement: File 1 — Additional data. [file Beilstein_J_Org_Chem-09-761-s001.pdf]

Supporting Information

for

Ring opening of 2-aza-3-borabicyclo[2.2.0]hex-5-ene, the Dewar form of 1,2-dihydro-1,2-azaborine: stepwise versus concerted mechanisms

Holger F. Bettinger* and Otto Hauler

Address: Institut für Organische Chemie, Universität Tübingen, Auf der Morgenstelle 18, 72072 Tübingen, Germany

Email: Holger F. Bettinger - holger.bettinger@uni-tuebingen.de

*Corresponding author

Additional data

Table of Contents

1. Cartesian coordinates of stationary points computed at CCSD(T)/TZ2P. S2

2. Cartesian coordinates of stationary points computed at CASSCF(6,6)/6-31G*. S3

3. Cartesian coordinates of dimers of **3** computed at SCS-RIMP2/def2-TZVP. S4

4. Geometries of dimers of **3**. S6

1. Cartesian coordinates of stationary points computed at CCSD(T)/TZ2P.

```
   12
2-Aza-3-bora-bicyclo[2.2.0]hexa-5-ene, 3
 C    -1.246668    -0.752826    -0.266716
 C    -1.379721     0.589622    -0.283038
 C    -0.103115     0.878375     0.515334
 B     1.281588     0.776253    -0.323082
 N     1.308130    -0.621550    -0.198095
 C     0.038228    -0.702867     0.562392
 H     0.089895    -1.244579     1.504911
 H    -0.188610     1.450443     1.436815
 H    -2.098676     1.252150    -0.747569
 H    -1.788040    -1.564332    -0.734096
 H     2.041542     1.509965    -0.868856
 H     1.812997    -1.393776    -0.595988


   12
1,2-Dihydro-1,2-Azaborine, 4
 C    -1.139809    -0.930717     0.000000
 B     0.293769    -1.439628     0.000000
 N     1.297479    -0.413701     0.000000
 C     1.010472     0.930276     0.000000
 C    -0.284936     1.369339     0.000000
 C    -1.363196     0.429445     0.000000
 H     1.849704     1.612874     0.000000
 H    -2.003040    -1.587172     0.000000
 H    -2.373915     0.825883     0.000000
 H    -0.479159     2.432402     0.000000
 H     0.656735    -2.574152     0.000000
 H     2.276990    -0.648047     0.000000


   12
MIN1
 N    -1.512141    -0.345668     0.394886
 B    -0.806361     1.080786    -0.056225
 C     0.678776     1.194200    -0.026993
 C     1.502574    -0.004725     0.178307
 C     0.783153    -1.132171    -0.036104
 C    -0.560588    -0.704455    -0.507544
 H    -1.145418    -0.550784     1.326124
 H    -1.571508     1.882445    -0.477638
 H     1.175707     2.127540    -0.282424
 H     2.525341     0.018970     0.536677
 H     1.018305    -2.145520     0.258510
 H    -0.806673    -0.630617    -1.562275


   12
MIN2
 C     0.784556     1.147003    -0.061634
 B    -0.707850     1.083538    -0.010237
 N    -1.472461    -0.250666     0.458336
 C    -0.612846    -0.665648    -0.527416
 C     0.689694    -1.155105     0.005175
 C     1.515394    -0.093947     0.166957
 H    -0.885877    -0.742588    -1.576817
 H     1.314972     2.049134    -0.355714
 H     2.531192    -0.138739     0.541344
 H     0.801428    -2.151024     0.412788
 H    -1.446351     1.916056    -0.430581
 H    -2.424163    -0.145555     0.116690


   12
TS3
 C    -0.325862     1.153348     0.148622
 C    -1.478313     0.307297    -0.294744
 C    -1.068539    -0.929269     0.023780
 C     0.282608    -0.606974     0.597942
 B     1.136665     0.845030    -0.137682
 N     1.384866    -0.638700    -0.336781
 H    -0.532628     1.992081     0.813153
 H     0.521681    -0.732238     1.646821
 H    -2.377877     0.646250    -0.791424
 H    -1.459908    -1.913474    -0.194989
 H     2.003430     1.557607     0.260915
 H     1.026701    -1.006744    -1.213988


   12
TS4
 C     0.270748     1.373246     0.062090
 C    -1.081069     0.844822    -0.111696
 C    -1.386917    -0.506650    -0.090383
 C    -0.152044    -1.113671     0.404456
```

```
 N     0.922836    -1.023898    -0.338438
 B     1.451269     0.503934     0.111539
 H     0.347763     2.453204     0.131331
 H     0.012846    -1.255399     1.475174
 H    -1.912974     1.540116    -0.183250
 H    -2.387803    -0.897076     0.001607
 H     2.620122     0.657685     0.216663
 H     0.616923    -0.894290    -1.306556
```

```
   12
TS5
 C     0.416558     1.158332    -0.148259
 B    -1.053027     0.889261     0.130380
 N    -1.361740    -0.554668     0.413899
 C    -0.332812    -0.624983    -0.585605
 C     0.998021    -0.985784     0.005804
 C     1.504081     0.230788     0.259613
 H    -0.572009    -0.773099    -1.633283
 H     0.649367     2.017531    -0.775507
 H     2.439067     0.510795     0.726731
 H     1.301287    -1.977118     0.313271
 H    -1.901537     1.620826    -0.276508
 H    -2.281864    -0.767212     0.047899
```

```
   12
TS6
 C    -0.877941    -1.136811    -0.121632
 B     0.571819    -1.243393    -0.019314
 N     1.472361     0.099937     0.403689
 C     0.729848     0.810781    -0.425596
 C    -0.626754     1.217229     0.042333
 C    -1.474732     0.171025     0.144728
 H     1.122006     1.152775    -1.385421
 H    -1.514578    -1.984801    -0.342059
 H    -2.496996     0.292720     0.485207
 H    -0.787988     2.228482     0.390674
 H     1.346325    -2.097875    -0.304452
 H     2.412631    -0.044973     0.046475
```

2. Cartesian coordinates of stationary points computed at CASSCF(6,6)/6-31G*.

```
   12
2-Aza-3-bora-bicyclo[2.2.0]hexa-5-ene, 3
 C    -0.239026     0.918960     0.387615
 C    -1.444453     0.257965    -0.268883
 C    -1.032307    -1.005711    -0.066603
 C     0.239297    -0.588145     0.650208
 B     1.106296     0.958365    -0.507401
 N     1.421072    -0.362784    -0.182772
 H    -0.413768     1.597682     1.212444
 H     0.436996    -0.972524     1.642306
 H    -2.287753     0.678905    -0.785735
 H    -1.401942    -1.965645    -0.378204
 H     1.667891     1.730137    -1.216633
 H     2.041544    -1.065493    -0.520004
```

```
   12
1,2-Dihydro-1,2-Azaborine, 4
 C    -0.852678     1.070677     0.000000
 C     0.842766    -1.207971     0.000000
 C    -0.519498    -1.334147     0.000000
 C    -1.378927    -0.180560     0.000000
 H    -1.466139     1.951869     0.000000
 H     1.438894    -2.105550     0.000000
 H    -0.987546    -2.305005     0.000000
 H    -2.446307    -0.301478     0.000000
 B     1.451558     0.201658     0.000000
 H     2.612786     0.470986     0.000000
 N     0.512335     1.263347     0.000000
 H     0.817242     2.212271     0.000000
```

```
   12
TS1
 C    -1.134097    -0.855982    -0.188232
 C     0.173402    -0.993628     0.517799
 C    -0.216708     1.209815     0.311219
 C    -1.393103     0.459388    -0.220718
 H     0.224975    -0.992247     1.590982
 H    -0.399295     2.030086     0.990084
 H    -2.256847     0.944565    -0.645899
 H    -1.672794    -1.658172    -0.660071
 B     1.228157     0.841494    -0.237425
 N     1.334612    -0.549837    -0.195340
```

```
 H     2.082556     1.590279    -0.602907
 H     1.959478    -1.188774    -0.635875
```

```
   12
TS2
 C    -1.134097    -0.855982    -0.188232
 C     0.173402    -0.993628     0.517799
 C    -0.216708     1.209815     0.311219
 C    -1.393103     0.459388    -0.220718
 H     0.224975    -0.992247     1.590982
 H    -0.399295     2.030086     0.990084
 H    -2.256847     0.944565    -0.645899
 H    -1.672794    -1.658172    -0.660071
 B     1.228157     0.841494    -0.237425
 N     1.334612    -0.549837    -0.195340
 H     2.082556     1.590279    -0.602907
 H     1.959478    -1.188774    -0.635875
```

3. Cartesian coordinates of dimers of **3** computed at SCS-RIMP2/def2-TZVP.

```
   24
DIM1
 C     3.213611    -0.226836     0.676286
 C     3.262779    -0.208884    -0.671777
 C     1.948674     0.513861    -0.848413
 C     1.924582     0.556086     0.728473
 H     1.923927     1.434477    -1.435003
 H     1.877788     1.485747     1.298276
 H     3.810715    -0.700118     1.448198
 H     3.950283    -0.647126    -1.388491
 C    -3.262779     0.208884     0.671777
 C    -1.948674    -0.513861     0.848413
 C    -1.924582    -0.556086    -0.728473
 C    -3.213611     0.226836    -0.676286
 H    -1.923927    -1.434477     1.435003
 H    -1.877788    -1.485747    -1.298276
 H    -3.950283     0.647126     1.388491
 H    -3.810715     0.700118    -1.448198
 N     0.701609    -0.296038     0.875723
 H     0.762572    -1.033289     1.571321
 N    -0.701609     0.296038    -0.875723
 H    -0.762572     1.033289    -1.571321
 B    -0.660503     0.490281     0.764822
 H    -0.659725     1.599924     1.233192
 B     0.660503    -0.490281    -0.764822
 H     0.659725    -1.599924    -1.233192
```

```
   24
DIM2
 C    -2.792348    -0.652449    -0.304267
 C    -2.769426     0.682613    -0.486689
 C    -1.260972     0.787305    -0.567844
 C    -1.287838    -0.779273    -0.385268
 H    -0.830920     1.243273    -1.456047
 H    -0.840072    -1.475653    -1.094049
 H    -3.570921    -1.371497    -0.071845
 H    -3.548299     1.438865    -0.486049
 C     1.675124    -0.714865    -1.416077
 C     2.006159    -0.898285     0.050147
 C     2.075607     0.674094     0.070244
 C     1.691612     0.634743    -1.387321
 H     2.897997    -1.481341     0.289060
 H     2.996605     1.183723     0.355571
 H     1.463983     1.409827    -2.109794
 H     1.473981    -1.425871    -2.211990
 N    -0.665772    -0.790627     0.979113
 H    -1.201436    -1.304764     1.669961
 N     0.989493     0.909658     1.087064
 H     1.299348     1.469582     1.871851
 B    -0.586027     0.880728     0.917492
 H    -1.104768     1.488226     1.817102
 B     0.883735    -0.740187     1.239829
 H     1.162500    -1.164685     2.330166
```

```
   24
DIM3
 C     1.357266    -0.515089    -1.504113
 C     1.579927    -1.066458    -0.112724
 C     2.017398     0.411427     0.220451
 C     1.698884     0.756047    -1.206883
 H     2.310236    -1.874612    -0.028663
 H     3.017403     0.613538     0.606718
 H     1.675250     1.695181    -1.748398
 H     1.020652    -0.987455    -2.420838
```

N	-0.982760	-0.700175	1.267297
H	-1.377944	-1.080383	2.118908
N	0.982760	0.700175	1.267297
H	1.377944	1.080383	2.118908
C	-1.357266	0.515089	-1.504113
C	-1.579927	1.066458	-0.112724
C	-2.017398	-0.411427	0.220451
C	-1.698884	-0.756047	-1.206883
H	-1.675250	-1.695181	-1.748398
H	-1.020652	0.987455	-2.420838
H	-2.310236	1.874612	-0.028663
H	-3.017403	-0.613538	0.606718
B	-0.579121	0.921219	1.187494
H	-0.948801	1.547786	2.146520
B	0.579121	-0.921219	1.187494
H	0.948801	-1.547786	2.146520

24
TS7

C	-3.535731	-0.579938	0.041315
C	-3.456900	0.761280	0.146282
C	-2.030310	0.863828	-0.368074
C	-2.133158	-0.707362	-0.527593
H	-1.827616	1.507269	-1.223598
H	-1.990484	-1.204985	-1.488880
H	-4.276967	-1.314534	0.335748
H	-4.134466	1.513247	0.537127
C	3.456911	-0.761259	-0.146313
C	2.030338	-0.863829	0.368086
C	2.133163	0.707362	0.527597
C	3.535719	0.579960	-0.041359
H	1.827677	-1.507273	1.223615
H	1.990513	1.204987	1.488887
H	4.134479	-1.513215	-0.537179
H	4.276933	1.314568	-0.335818
N	-1.054975	-0.911438	0.462291
H	-0.935627	-1.755760	1.003884
N	1.054945	0.911416	-0.462253
H	0.935561	1.755736	-1.003843
B	0.924781	-0.498268	-0.752718
H	0.460953	-1.033265	-1.711210
B	-0.924781	0.498244	0.752752
H	-0.460958	1.033230	1.711254

4. Geometries of dimers of **3**.

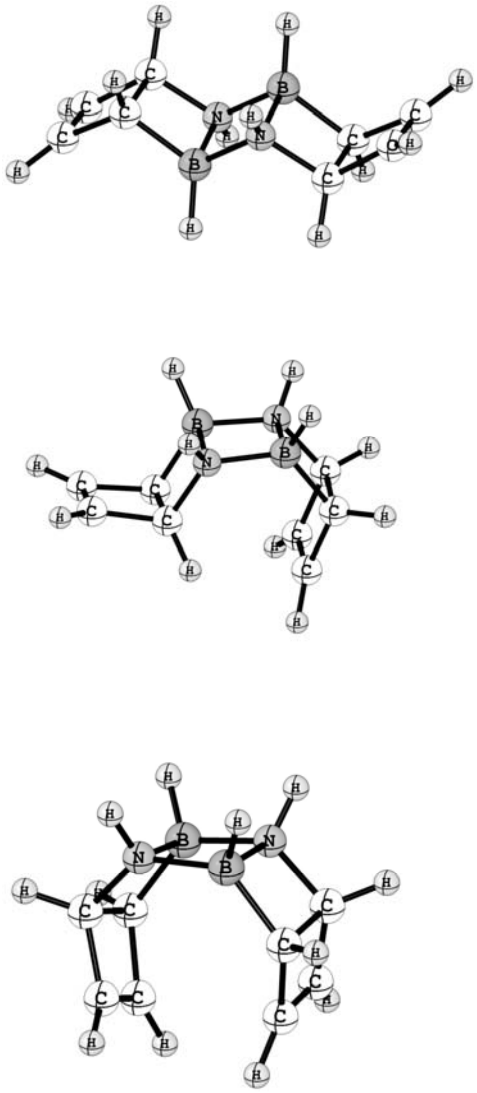

	E_{rel}[kcal mol^{-1}]
**DIM1**	0
**DIM2**	10.6
**DIM3**	23.2

Figure S1: Computed geometries of dimers of **3** and energy relative to the most stable DIM1 as computed at the SCS-RIMP2/def2-TZVP level of theory.
